# Effectiveness of Liquid-Liquid Extraction, Solid Phase Extraction, and Headspace Technique for Determination of Some Volatile Water-Soluble Compounds of Rose Aromatic Water

**DOI:** 10.1155/2017/4870671

**Published:** 2017-07-16

**Authors:** Hale Seçilmiş Canbay

**Affiliations:** Department of Bioengineering, Faculty of Engineering and Architecture, Mehmet Akif Ersoy University, 15030 Burdur, Turkey

## Abstract

Steam distillation is used to isolate scent of rose flowers. Rose aromatic water is commonly used in European cuisine and aromatherapy besides its use in cosmetic industry for its lovely scent. In this study, three different sampling techniques, liquid-liquid extraction (LLE), headspace technique (HS), and solid phase extraction (SPE), were compared for the analysis of volatile water-soluble compounds in commercial rose aromatic water. Some volatile water-soluble compounds of rose aromatic water were also analyzed by gas chromatography mass spectrometry (GCMS). In any case, it was concluded that one of the solid phase extraction methods led to higher recoveries for 2-phenylethyl alcohol (PEA) in the rose aromatic water than the liquid-liquid extraction and headspace technique. Liquid-liquid extraction method provided higher recovery ratios for citronellol, nerol, and geraniol than others. Ideal linear correlation coefficient values were observed by GCMS for quantitative analysis of volatile compounds (*r*^2^ ≥ 0.999). Optimized methods showed acceptable repeatability (RSDs < 5%) and excellent recovery (>95%). For compounds such as *α*-pinene, linalool, *β*-caryophyllene, *α*-humulene, methyl eugenol, and eugenol, the best recovery values were obtained with LLE and SPE.

## 1. Introduction

Aromatic waters are clear and saturated solutions of volatile oils, aromatic or volatile substances, and some water-soluble compounds of essential oil. Aromatic waters are generally produced by distillation of aromatic plants such as rose, thyme, and rosemary. Water acquired by concentration of steam during steam distillation to isolate the scent of aromatic plant flowers includes low amounts of essential oil. Rose aromatic water is liquid preparation obtained by hydrosol portion of the distillate of fresh rose petals and contains aromatic compounds in the form of either solution or suspended particles [1–4].

Rose aromatic water is a commercially important commodity since it is commonly used in European cuisine and aroma therapy. Due to its fragrance, rose aromatic water is used in cosmetic industry, food flavoring, soaps, and toiletry. It is also used in traditional medicine as antiseptic facial tonic, fever reducer, cooling assist, pain killer, astringent, mild laxative, and antibacterial and in treatment of sore throat, enlarged tonsils, cardiac troubles, eye diseases, gall stones, and gut troubles [3, 5–9].

In water distillation method, rose aromatic water contains very low amounts of (below 0.1%) essential oil and its main component is phenylethyl alcohol [4, 10–16]. Double distillation method is used as a traditional method. In this method, rose aromatic water is obtained in the final step of rose oil production, which is retained in large-capacity copper/stainless steel distillation devices [17].

Due to the complexity of rose aromatic water and relatively low concentration of some terpenes, its analyses require isolation/preconcentration steps. The low concentration of volatile compounds makes enrichment necessary as the basis for identification and quantification, and among them liquid-liquid extraction (LLE), solid phase extraction (SPE), solid phase microextraction (SPME), simultaneous distillation extraction (SDE), supercritical fluid extraction (SFE), headspace solid phase microextraction (HS-SPME), solid phase extraction (SPE), and stir bar sorptive extraction (SBSE) have been extensively studied [2–4, 10–16, 18–36].

This study is aimed at selecting the best extraction technique for studying the volatile composition of rose water, and LLE, SPE, and HS technique were used for quantitative determination of volatile compounds of aromatic rose water. Numerous studies have been carried out on chemical composition of rose aromatic water [3, 4, 10–16, 19] from different countries but analytical values (extraction efficiency, detection limit, etc.) were not included in these studies. To our knowledge, this present study is the first involving these parameters.

## 2. Materials and Methods

### 2.1. Chemicals and Reagents

Methanol (HPLC grade), n-hexane (HPLC grade), n-pentane (HPLC grade), chloroform (GC grade), dichloromethane (HPLC grade), and ethyl acetate (HPLC grade) were obtained from Merck (Germany) and Sigma Aldrich (USA). Sep Pak Plus C18 cartridges (Waters, USA) and Isolute ENV+ (Biotage, USA) were used for solid phase extraction. Chemical standards of *α*-pinene, linalool, *α*-humulene (*α*-caryophyllene), nerol, 2-phenylethyl alcohol (PEA), *β*-caryophyllene, citronellol, geraniol, methyl eugenol, and eugenol were supplied from Sigma Aldrich (USA) while NaCl was purchased from Merck (Germany).

### 2.2. Aromatic Rose Water Samples

Commercial rose water samples were purchased from a local store.

### 2.3. Isolation and Preconcentration Techniques

Three different methods were used for the isolation and concentration of volatile compounds from rose aromatic water as explained below.

#### 2.3.1. Liquid-Liquid Extraction Procedure

The volatile components were extracted by the methods of Hernanz et al. [33] and Cabredo-Pinillos et al. [34]. Briefly, 200 mL of sample containing 4 g of NaCl was placed in a 250 mL glass flask. The extraction was performed with 5 mL of chloroform, dichloromethane, n-hexane, and ethyl acetate. The flasks were introduced into the ultrasonic bath (Bandelin Sonorex, Germany) and sonicated for 30 min at 25°C. The organic layer was then separated via pipetting. All samples were extracted in duplicate. Finally, 1 *μ*L of aliquots was injected into a GCMS system.

#### 2.3.2. Solid Phase Extraction Procedure

Two different solid phase cartridges (Sep Pak Plus C18 cartridges and Isolute ENV+) were tested for the isolation and concentration of volatile compounds from rose aromatic water (Table 1). The solid phase extractions were carried out in a Visiprep SPE vacuum manifold (12-port model) from Supelco (Supelco, USA). Solid phase extractions were performed according to the method developed by López et al. [20] and Piñeiro et al. [21]. Cartridges were placed in the manifold system and activated with 4 mL dichloromethane, 4 mL of methanol, and finally rinsing 4 mL of water. Then 100 mL rose aromatic water samples were passed through the cartridges by vacuum manifold, after which the sorbents were dried. Volatile compounds were eluted from the cartridges using an organic solvent (n-pentane, n-hexane, dichloromethane, and methanol).

#### 2.3.3. Headspace Extraction

Isolation and preconcentration of volatile compounds were achieved using a 7697A headspace sampler apparatus (Agilent, USA). 4 mL volume of sample was used for analysis.  Table 2 shows the headspace sampler setup parameters for the headspace sampler apparatus. The headspace transfer line was directly passed through the 7890A GC injector port and connected to the GC CP-Wax 52 CB column using a universal capillary column connector.

### 2.4. Standard Solutions, Calibration Curves, and Recovery Studies

Stock standard solutions of 10 mg/mL of each compound were prepared in methanol and stored at −20°C. In both cases, different working standard solutions were prepared by dilution in the same solvent. Six concentrations were used for calibration curves of volatile compounds. The average recoveries of the analytes were determined by comparing the peak areas obtained from each volatile compound.

### 2.5. Chromatography and Apparatus

Apparatus GCMS was Agilent 7890A gas chromatograph equipped with a 5975 mass detector (MSD), a 7693B automatic sampler, and a MSDCHEM (Agilent, USA) data system. Analytes were separated in a fused silica capillary column CP-Wax 52 CB stationary phase (50 m × 0.25 mm; film thickness 0.2 *μ*m) (Agilent, USA). Oven temperature program was as follows: initial temperature 60°C, held for 1 min, increased to 220°C at 2°C/min, and held at 20 min. The carrier gas (helium) flow rate was 15 psi. Splitless injection of a 1 *μ*L volume was carried out at 250°C. Temperature of the detector was 250°C. MSD conditions were ion source temperature, 230°C; electron energy, 70 eV; mass scan range, 30–500 amu. The same system and temperature program were used in HS analysis. A 7697A headspace sampler was used instead of automatic sampler [3].

### 2.6. Statistical Analyses and Validation Procedures

Limit of detection (LOD), limit of quantification (LOQ), linearity of calibration, intraday and interday accuracy, precision, and recovery were estimated for the validation of this method. Six concentrations of standard solutions were prepared. Each standard solutions (volatile compounds) concentration was measured in five replicates. Calibration curves for the studied volatile compounds were preprepared by plotting peak areas versus concentrations for GCMS and HS-GCMS. We defined the LOD was defined as three times the background noise of the chromatographic instrument. The extraction recovery was determined by spiking blank rose water with each compound (standard addition method) in three replicates; they were extracted as previously described. Standard solutions of target compounds were analyzed five times in a day and once a day on three consecutive days for intraday and interday precisions, respectively.

## 3. Results and Discussion

Ten volatile water-soluble compounds in rose aromatic water were used as target compounds during the LLE, SPE, and HS method development: *α*-pinene, linalool, *α*-humulene, nerol, PEA, *β*-caryophyllene, citronellol, geraniol, methyl eugenol, and eugenol.

### 3.1. Optimization of SPE and Eluting Solvent

Two different kinds of solid phase sorbents were studied, one of them with reversed solid phase (C18) and the other one with hydroxylated polystyrene-divinyl benzene copolymer (ENV+) solid phase. 2 mL of each eluting solvent was used to recover terpenoid ingredients from the solid phase sorbents. n-Pentane and n-hexane were the worst sorbents eluting solvents. Recoveries < 50% were obtained for all volatile water-soluble compounds for two kinds of solid phase sorbents. Methanol was selected as the best eluting solvent for compound elution from the Sep Pak Plus C18 cartridge. Also dichloromethane was the best eluting solvent for volatile water-soluble compounds elution from the Isolute ENV+. Piñeiro et al. [21] studied the effectiveness of four different eluting solvents (n-pentane, methanol, dichloromethane, and ethanol) for the SPE of wine terpenoids and concluded that the highest isolation and concentration efficiency was achieved by using dichloromethane as the eluting solvent.

### 3.2. Optimization of LLE and Extraction Solvent

Complete extraction from rose aromatic water was achieved by using chloroform, dichloromethane, ethyl acetate, and n-hexane. LLE of rose aromatic water with chloroform, dichloromethane, and ethyl acetate provided target compounds in rose aromatic water extract in quantitative determination. n-Hexane was worst LLE solvents.

Agarwal et al. [4] studied the effectiveness of different eluting solvents (dichloromethane, chloroform, hexane, and benzene) for the LLE of rose water terpenoids and concluded that the highest isolation and optimum results were achieved by using dichloromethane as extraction solvent.

### 3.3. Linearity of Calibration Curves and Limits of Detection and Quantification

The retention time (*R*_*t*_), linearity (*r*^2^), LOD, and LOQ were summarized in Table 3. A regression equation was obtained with good linearity (*r*^2^ ≥ 0.999) for GCMS and for HS-GCMS.

The *r*^2^ values ≥0.999 for target compounds. Lei et al. [15] reported the PEA in rose water and the acquired *r*^2^ value was 1.0000. Piñeiro et al. [21] studied SPE methods and found *r*^2^ values were >0.999. Vila et al. [35] reported volatile compounds in wine and linear correlation coefficients were ≥0.994. Won et al. [36] indicated PEA values in Bulgarian rose and Provence lavender oil and *r*^2^ value for PEA was 0.9814.

The LOD values were between 0.360 and 1.600 *μ*g/L for studied compounds for GCMS and between 1.143 and 4.650 *μ*g/L for studied compounds for HS-GCMS. The relatively low values indicated that the method possessed good sensitivity. In the HS-GCMS technique, the LOD values were slightly higher because the noise ratio was slightly higher than the GCMS technique. Piñeiro et al. [21] used SPE methods and obtained LOD values between 0.33 and 3.37 *μ*g/L. Sánchez-Palomo et al. [27] evaluated the LLE, SPE, and SDE methods, and the obtained LOD values were within 0.01–0.02 *μ*g/L, 0.02–0.04 *μ*g/L, and 0.01–0.08 *μ*g/L, respectively. Won et al. [36] reported PEA values in Bulgarian rose and Provence lavender oil and LOD value for PEA was 0.77 ng/mL.

Precision and repeatability are summarized in Tables 4 and 5. The intraday and interday relative standard deviations (RSDs) for target compounds were within 0.10%–0.23% and 0.13%–0.24%, respectively, for GCMS and within 0.29%–0.43% and 0.32%–0.47%, respectively, for HS-GCMS. The RSD values were less than 5% in all experiments. Lei et al. [15] studied the PEA in rose water and obtained intraday and interday RSDs of PEA 0.15% and 0.19%, respectively.

### 3.4. Recoveries

Recovery test was carried out using the method of standard addition. Rose water samples were spiked with three different concentrations of standard solutions and analyzed. The recovery of PEA ranged from 32.22% to 69.45%, with RSDs less than 5.00% for LLE. Recovery values ranged from 25.76 to 98.86% for chloroform as an extraction solvent while they varied from 32.07 to 98.81% for dichloromethane. For ethyl acetate, the range of recovery values was between 57.49 and 95.29%, which was generally higher than that of n-hexane (32.71 to 67.35%) (Table 6).

When the C18 cartridge was used, the recovery values were between 80.44 and 99.75%. For ENV+ recoveries ranged from 32.96 to 87.22%. For HS-GCMS recovery ranged between 33.53 and 116.90% (Table 7).

Lei et al. [15] reported that recovery values were within 99.3–101.0% for PEA in rose water. Piñeiro et al. [21] found that recovery values were within 96.8–100.8% for optimized method. Hernanz et al. [33] evaluated the effectiveness of different extraction methods (LLE and SPE), and the recovery values for PEA, linalool, citronellol, nerol, and geraniol were 92.6%, 109.6%, 97.2%, 97%, and 99.3% for LLE 1, 18.6%, 19.7%, 19.4%, 19.3%, and 20.7% for LLE 2, and 96.5%, 98.7%, 88.9%, 88.2%, and 97.4% for SPE, respectively.

### 3.5. Chemical Profiles of Rose Aromatic Water

The predominant components of rose water volatiles are PEA, citronellol, geraniol, nerol, and methyl eugenol [3, 4, 10–16, 19]. In addition to these components, *α*-pinene, linalool, *β*-caryophyllene, and *α*-humulene components were also included in our study. These components are also found in rose oil and rose water [3, 4, 10–19]. In rose aromatic water samples, PEA (1677.38 ± 0.14 *μ*g/g), citronellol (418.21 ± 0.23 *μ*g/g), nerol (183.77 ± 0.44 *μ*g/g), and geraniol (443.34 ± 0.12 *μ*g/g) were found as main compounds followed by *α*-pinene (63.62 ± 0.88 *μ*g/g), linalool (73.57 ± 0.38 *μ*g/g), *α*-humulene (22.93 ± 0.30 *μ*g/g), eugenol (66.25 ± 0.52 *μ*g/g), and methyl eugenol (55.10 ± 0.28 *μ*g/g). *β*-Caryophyllene was not detected in rose aromatic water samples.

## 4. Conclusions

This study was carried out to quantify the volatile water-soluble compounds of rose aromatic water samples using different isolation and preconcentration techniques. The recoveries obtained using samples spiked with standard target compounds ranged within 80.44 and 99.75% for C18 cartridge, 32.96 and 87.22% for ENV+, 33.53 and 116.90% for HS, 25.76 and 98.86% for chloroform, 32.07 and 98.81% for dichloromethane, 57.49 and 95.29% for ethyl acetate, and 32.71 and 67.35% for n-hexane. The RSD values were less than 5% in all experiments. The method showed good recoveries and high repeatabilities. PEA was the main volatile water-soluble constituent of aromatic rose water.

The SPE method is a faster technique than LLE and HS but the HS technique is achieved without any solvent. Different solvents such as chloroform, dichloromethane, ethyl acetate, n-pentane, and n-hexane are used in SPE and LLE techniques, which may cause contamination of the environment at different levels.

## Figures and Tables

**Table 1:** Main properties of solid phase cartridges used in isolation and concentration.

	Chemical description of solid phase sorbent	Supplier	Amount of solid phase cartridge (mg)
Sep Pak Plus C18	Reversed phase	Waters	360
Isolute ENV+	Hydroxylated polystyrene-divinyl benzene copolymer	Biotage	500

**Table 2:** Headspace sampler setup parameters.

Parameter	Working condition
Oven temperature	75°C
Loop temperature	90°C
Transfer line temperature	100°C
Vial pressure	0.23 psi
Vial flow	20 mL/min
Carrier pressure	15 psi

**Table 3:** Retention time (*R*_*t*_) and performance characteristic obtained by GCMS and HS-GCMS.

Compounds	*R*_*t*_ (min)	*r* ^2^		LOD_GCMS_ (*µ*g/L)	LOQ_GCMS_ (*µ*g/L)	LOD_HS-GCMS_ (*µ*g/L)	LOQ_HS-CMS_ (*µ*g/L)
		GCMS	HS-GCMS				
*α*-Pinene	4.3	0.9997	0.9990	1.240	4.092	3.750	12.375
Linalool	9.8	0.9999	0.9990	0.360	1.188	1.143	3.772
*β*-Caryophyllene	10.4	0.9999	0.9990	0.680	2.244	2.100	6.930
*α*-Humulene	11.0	0.9996	0.9990	0.720	2.376	2.250	7.425
Citronellol	11.4	0.9995	0.9990	0.380	1.254	1.210	3.993
Nerol	11.8	0.9994	0.9990	1.000	3.300	3.600	11.880
Geraniol	12.2	0.9990	0.9990	1.600	5.280	4.650	15.25
PEA	13.1	0.9990	0.9990	1.000	3.300	2.780	9.174
Methyl eugenol	14.4	0.9996	0.9990	0.780	2.574	2.455	8.102
Eugenol	17.6	0.9999	0.9990	0.600	1.980	1.890	6.237

**Table 4:** Intraday and interday precisions for GCMS.

Compounds	Precision (RSD, %)			Precision (RSD, %)		
	Intraday (mg/L)			Interday (mg/L)		
	2.50	5.00	10.00	2.50	5.00	10.00
*α*-Pinene	2.48 (0.12)	5.11 (0.10)	9.98 (0.10)	2.45 (0.15)	4.98 (0.14)	9.95 (0.13)
Linalool	2.52 (0.15)	5.05 (0.15)	10.08 (0.14)	2.51 (0.17)	4.99 (0.16)	9.98 (0.16)
*β*-Caryophyllene	2.45 (0.13)	5.01 (0.13)	9.95 (0.15)	2.43 (0.17)	4.98 (0.17)	9.96 (0.16)
*α*-Humulene	2.43 (0.15)	5.05 (0.15)	9.98 (0.13)	2.42 (0.18)	4.96 (0.16)	9.94 (0.16)
Citronellol	2.52 (0.11)	5.03 (0.10)	10.02 (0.13)	2.49 (0.18)	4.98 (0.18)	9.95 (0.18)
Nerol	2.48 (0.15)	4.95 (0.14)	9.95 (0.15)	2.44 (0.18)	4.95 (0.18)	9.93 (0.17)
Geraniol	2.47 (0.13)	5.02 (0.14)	9.95 (0.13)	2.48 (0.15)	4.98 (0.15)	9.95 (0.15)
PEA	2.51 (0.14)	5.05 (0.13)	10.02 (0.12)	2.53 (0.17)	4.97 (0.16)	9.97 (0.17)
Methyl eugenol	2.46 (0.20)	4.96 (0.19)	9.98 (0.20)	2.48 (0.22)	4.96 (0.20)	9.95 (0.20)
Eugenol	2.49 (0.23)	5.11 (0.20)	10.08 (0.21)	2.45 (0.24)	5.05 (0.22)	9.99 (0.23)

**Table 5:** Intraday and interday precisions for HS-GCMS.

Compounds	Precision (RSD, %)			Precision (RSD, %)		
	Intraday (mg/L)			Interday (mg/L)		
	2.50	5.00	10.00	2.50	5.00	10.00
*α*-Pinene	2.42 (0.32)	4.88 (0.30)	10.21 (0.31)	2.40 (0.35)	5.89 (0.36)	10.18 (0.35)
Linalool	2.47 (0.36)	5.11 (0.35)	9.88 (0.35)	2.45 (0.38)	4.95 (0.38)	9.95 (0.36)
*β*-Caryophyllene	2.47 (0.37)	4.97 (0.35)	9.85 (0.37)	2.46 (0.40)	4.95 (0.39)	9.84 (0.39)
*α*-Humulene	2.44 (0.38)	4.96 (0.38)	9.88 (0.37)	2.44 (0.41)	4.94 (0.42)	9.85 (0.40)
Citronellol	2.45 (0.30)	4.85 (0.29)	9.85 (0.30)	2.42 (0.33)	4.83 (0.32)	9.88 (0.32)
Nerol	2.52 (0.35)	5.08 (0.34)	9.86 (0.35)	2.49 (0.37)	4.95 (0.36)	9.85 (0.36)
Geraniol	2.48 (0.31)	4.94 (0.31)	9.91 (0.30)	2.44 (0.34)	4.90 (0.33)	9.95 (0.33)
PEA	2.45 (0.32)	4.89 (0.32)	9.98 (0.33)	2.47 (0.35)	4.87 (0.34)	9.95 (0.34)
Methyl eugenol	2.53 (0.41)	5.10 (0.42)	10.13 (0.41)	2.55 (0.45)	4.99 (0.43)	10.03 (0.43)
Eugenol	2.49 (0.43)	5.11 (0.43)	10.08 (0.42)	2.45 (0.47)	5.05 (0.45)	9.99 (0.46)

**Table 6:** LLE average recovery.

Compounds	Average percent recovery and RSD values (%) Extraction solvent type			
	Chloroform	Dichloromethane	Ethyl acetate	n-Hexane
*α*-Pinene	31.43 (1.10)	32.07 (0.98)	57.49 (0.93)	32.71 (0.87)
Linalool	25.76 (1.35)	97.86 (0.78)	95.29 (0.81)	61.01 (1.23)
*β*-Caryophyllene	90.72 (0.92)	94.22 (0.81)	81.28 (0.98)	61.96 (1.35)
*α*-Humulene	90.10 (0.85)	84.25 (1.11)	80.65 (0.98)	46.40 (1.42)
Citronellol	97.15 (0.81)	87.50 (0.99)	87.39 (1.01)	61.76 (1.19)
Nerol	95.48 (1.04)	97.47 (0.85)	85.56 (1.05)	50.96 (1.17)
Geraniol	98.86 (0.83)	86.61 (0.88)	95.24 (0.85)	52.29 (1.10)
PEA	69.45 (0.95)	65.57 (1.22)	67.38 (1.25)	32.22 (1.55)
Methyl eugenol	84.58 (0.95)	94.63 (0.80)	77.67 (1.18)	67.35 (1.21)
Eugenol	88.22 (0.90)	98.81 (0.75)	91.80 (0.95)	49.36 (1.30)

**Table 7:** SPE average recovery.

Compounds	Average percent recovery and RSD values (%) Extraction type		
	C18	ENV+	HS-GCMS
*α*-Pinene	91.45 (0.78)	32.96 (1.20)	60.11 (2.13)
Linalool	86.44 (0.98)	76.61 (0.90)	61.48 (2.25)
*β*-Caryophyllene	80.44 (0.75)	59.56 (0.93)	59.03 (2.35)
*α*-Humulene	85.85 (0.92)	59.03 (1.02)	45.32 (3.12)
Citronellol	85.69 (0.96)	75.87 (0.91)	58.21 (2.01)
Nerol	86.46 (0.96)	72.71 (0.95)	46.39 (2.41)
Geraniol	85.73 (0.90)	73.04 (1.05)	48.00 (2.39)
PEA	99.75 (0.83)	80.33 (0.85)	33.53 (2.17)
Methyl eugenol	84.20 (0.96)	85.22 (1.00)	116.90 (1.94)
Eugenol	85.01 (0.99)	87.22 (0.96)	83.49 (1.96)
